# Random forest-based prediction of intracranial hypertension in patients with traumatic brain injury

**DOI:** 10.1186/s40635-024-00643-6

**Published:** 2024-07-02

**Authors:** Jun Zhu, Yingchi Shan, Yihua Li, Xuxu Xu, Xiang Wu, Yajun Xue, Guoyi Gao

**Affiliations:** 1 Department of Neurosurgery, Shanghai General Hospital, Shanghai Jiao Tong University School of Medicine, Shanghai, 201600 China; 2 Department of Neurosurgery, Xinhua Hospital Affiliated to Shanghai Jiao Tong University School of Medicine, Shanghai, 200092 China; 3 Department of Neurosurgery, Minhang Hospital Fudan University, Shanghai, 201199 China; 4 Department of Neurosurgery, Beijing Tiantan Hospital, Capital Medical University, Beijing, 100070 China; 5 Neurotrauma Laboratory, Beijing Neurosurgical Institute, Capital Medical University, Beijing, 100070 China

**Keywords:** Traumatic brain injury, Intracranial hypertension, Random forest, Complexity

## Abstract

**Background:**

Treatment and prevention of intracranial hypertension (IH) to minimize secondary brain injury are central to the neurocritical care management of traumatic brain injury (TBI). Predicting the onset of IH in advance allows for a more aggressive prophylactic treatment. This study aimed to develop random forest (RF) models for predicting IH events in TBI patients.

**Methods:**

We analyzed prospectively collected data from patients admitted to the intensive care unit with invasive intracranial pressure (ICP) monitoring. Patients with persistent ICP > 22 mmHg in the early postoperative period (first 6 h) were excluded to focus on IH events that had not yet occurred. ICP-related data from the initial 6 h were used to extract linear (ICP, cerebral perfusion pressure, pressure reactivity index, and cerebrospinal fluid compensatory reserve index) and nonlinear features (complexity of ICP and cerebral perfusion pressure). IH was defined as ICP > 22 mmHg for > 5 min, and severe IH (SIH) as ICP > 22 mmHg for > 1 h during the subsequent ICP monitoring period. RF models were then developed using baseline characteristics (age, sex, and initial Glasgow Coma Scale score) along with linear and nonlinear features. Fivefold cross-validation was performed to avoid overfitting.

**Results:**

The study included 69 patients. Forty-three patients (62.3%) experienced an IH event, of whom 30 (43%) progressed to SIH. The median time to IH events was 9.83 h, and to SIH events, it was 11.22 h. The RF model showed acceptable performance in predicting IH with an area under the curve (AUC) of 0.76 and excellent performance in predicting SIH (AUC = 0.84). Cross-validation analysis confirmed the stability of the results.

**Conclusions:**

The presented RF model can forecast subsequent IH events, particularly severe ones, in TBI patients using ICP data from the early postoperative period. It provides researchers and clinicians with a potentially predictive pathway and framework that could help triage patients requiring more intensive neurological treatment at an early stage.

**Supplementary Information:**

The online version contains supplementary material available at 10.1186/s40635-024-00643-6.

## Background

Traumatic brain injury (TBI) is the leading cause of injury-related disability and death worldwide [1]. Since primary brain injury after TBI is inevitable, secondary brain injury has been the focus of most studies to identify potential therapeutic targets [2, 3]. Neurocritical care is central to minimizing secondary brain injury [4], of which elevated intracranial pressure (ICP) is a critical mediator [5]. Elevated ICP is a common complication of moderate-to-severe TBI and is strongly associated with poor prognosis [6–8]. Therefore, the cornerstone of neuroprotective management is monitoring ICP and treating intracranial hypertension (IH) with strategies that escalate in intensity based on elevated ICP [8–10].

Currently, IH clinical management relies on invasive measurement of ICP through external ventricular drainage or intraparenchymal probes [9, 11]. Prolonged and sustained intracranial hypertension should be avoided to protect brain tissue from secondary deterioration [12]. Due to the pathophysiology of the intracranial pressure–volume curve [13], ICP rises rapidly when intracranial contents approach intracranial volume. Consequently, current treatment for IH is reactive universally, making it challenging for clinicians to identify patients whose ICP will rapidly progress to dangerous levels [14]. Therefore, there is an urgent need for early clinical prediction models to anticipate the onset of IH and empower clinicians to intervene before subsequent adverse events. Recent clinical IH prediction models have achieved promising results by applying advanced signal processing and machine learning techniques [14–18]. However, a readily deployed bedside software solution is currently in need of improvement, and early warning of upcoming IH remains a significant and feasible research objective.

Due to multiple linear and nonlinear correlation determinants, ICP is a highly complex parameter influenced by various intracranial and extracranial factors [19–21]. Complexity-related nonlinear signal features, such as approximate entropy, are now recognized as potential summaries of homeostatic integrity [22, 23]. ICP complexity has been shown to decrease at the onset of IH [24, 25], and it has become a valuable prognostic predictor of TBI outcomes [26, 27]. Therefore, in this study, we incorporated complexity features based on ICP and cerebral perfusion pressure (CPP) into the IH prediction model to provide a comprehensive assessment of the intracranial condition in TBI patients. In addition, we included the pressure reactivity index (PRx), which contributes to individualized therapy, and the cerebrospinal fluid compensatory reserve index (RAP) as “linear” signal features together with ICP and CPP due to their established derivations.

Random forest (RF), a tree-based nonlinear algorithm, has shown superiority in machine learning algorithms, such as solving the overfitting problem of decision trees [28]. RF was identified as the best classifier in a study evaluated against 179 classifiers available today [29]. In the realm of TBI, RF has demonstrated its predictive capabilities for the prognosis of intracranial injury [30, 31].

In this study, we sought to develop prediction models for IH using RF classifiers by incorporating demographic characteristics (age, sex, and Glasgow Coma Scale score) with linear and nonlinear features. Specifically, we utilized ICP-related data from the initial 6-h period and extracted target features for predicting upcoming IH events during the postoperative monitoring period, aiming to support the prophylactic management of acute IH.

## Methods

### Patient selection and data acquisition

This study analyzed a prospective dataset of TBI patients admitted to the Department of Neurosurgery at Shanghai General Hospital between January 2021 and December 2021. The study protocol complied with the ethical guidelines of the Declaration of Helsinki and was approved by the institutional review. Informed consent of participants or proxies was adequately assured in the prospective data collection. As part of the intensive care unit (ICU) prospective cohort recruitment, demographics, injuries, and high-frequency digital signals from ICU monitoring were recorded for all patients. For this study, age, sex, and Glasgow Coma Scale (GCS) score at admission were extracted from the database. Patients underwent emergency surgery, including borehole drainage, cranial hematoma removal, or even decompressive craniectomy. ICP was monitored using a ventricular probe (Integra LifeSciences, Princeton, NJ) which was placed as the departmental clinical protocol. Invasive arterial blood pressure (ABP) was recorded via a bedside radial artery catheter with the transducer at the heart level. All signals were continuously collected using the data processing tool (Neuro Critical Care Data Processing System, Hunan Haotongxiangju Medical Technology) with data transmitted to an online server (https://www.neumatic.cn).

There were 102 patient recordings available. The total length of data recording varied between patients from 6 to 370 h. To observe the occurrence of IH, we focused on patients who had time for the disease process to evolve. As such, 93 patients with at least 18 h of recording of ICP, mean arterial pressure (MAP), and CPP were selected. Also, patients (*n* = 8) with numerous missing data and signal artifacts due to technical problems during recording, re-recording, or interruption were excluded. In addition, patients with severe TBI with mean ICP values consistently exceeding 22 mmHg in the first 6 h (*n* = 16) were excluded. As a result, 69 patients entered our analysis. All patients received analgesia, sedation, and mechanical ventilation during their stay in the intensive care unit and were managed according to a CPP-oriented protocol. Treatment options include head elevation, sedation, hyperosmolar therapy, hyperventilation, cerebrospinal fluid drainage, and even decompressive craniectomy to keep CPP above 60–70 mmHg.

Data from the first 6 h after the start of ICP stabilization monitoring were extracted for analysis. To suppress the pulse and respiratory waves and to focus entirely on the slow fluctuations of ICP, the data were resampled by averaging over 12-s epochs. Subsequently, a moving average filter, with the span set to 4, was used to remove sharp noise and smooth the data. The pressure amplitude correlation index (RAP) was calculated as the moving Pearson correlation coefficient between ICP pulse amplitude and ICP, representing the cerebrospinal compensatory reserve. In addition, the pressure reactivity index (PRx), a moving Pearson correlation between ICP and MAP, was calculated as a measure of cerebral autoregulation.

### Nonlinear signal information-based features

Sample entropy (SampEn) and Lempel–Ziv complexity (Lzc) were used as indicators of the nonlinear complexity of ICP (ICP_SampEn and ICP_Lzc) and CPP (CPP_SampEn and CPP_Lzc) sequences. SampEn, an improved version of approximate entropy, was computed according to Delgado-Bonal using R code [23]. For the two input parameters, m (embedding dimension) and r (noise filter), we adopted the traditional *m* = 2 and *r* = 0.2 standard deviation [32]. Lzc describes the rate at which new patterns emerge in the time series [33]. The Lzc algorithm is applied to symbolic sequences [34], so we binarized the time series before analysis. A detailed description of the Lzc algorithm is available in the original article [33]. Briefly, The Lzc uses a stepwise strategy to quantify the number of non-redundant patterns in a signal. At each time point of the signal, the complexity counter increases if the next symbol of the signal introduces a pattern that has never been observed before or cannot be replicated from previous segments [34].

### Prediction task

Intracranial hypertension (IH) prediction was defined as a binary classification task. The Brain Trauma Foundation (BTF) recommends treatment of ICP > 22 mmHg, which is an accepted threshold for IH [9, 35], due to values beyond this threshold are associated with increased mortality [35]. Although guidelines have not established a minimum duration of IH associated with harm to the brain, there is evidence of worse outcomes after 5 min of elevated ICP [36]. In this study, the primary outcome measure was IH, defined as ICP > 22 mmHg for > 5 min, and the secondary outcome measure was severe IH (SIH), defined as ICP > 22 mmHg for > 1 h, representing a more malignant intracranial condition.

### Model development

The dataset was randomly divided into 70% for training and 30% for testing using the “createDataPartition” function in the “caret” package while ensuring a balanced ratio of outcome metrics. Baseline characteristics (age, sex, and GCS score), linear features (ICP, CPP, PRx, and RAP), and nonlinear features of ICP and CPP (SampEn and Lzc) were included for analysis.

Random forest (RF) is a supervised machine-learning algorithm that generates multiple decision trees on a bootstrap basis and merges them to obtain more accurate and stable predictions. To select the most relevant features, Recursive Feature Elimination (RFE) based on the RF was applied to the training sets. Briefly, RFE is the iterative construction of a model by removing the least relevant features from the current feature set and repeating this step [37]. Cross-validation is combined to find the optimal number of features to avoid overfitting. The hyperparameters of the RF classifier were then optimally tuned using a grid search. To evaluate the presence or absence of multicollinearity between features, the variance inflation factor (VIF) for the selected features was calculated. Multicollinearity is usually considered to exist when the VIF exceeds the threshold of 5–10 [38]. Test sets were used to evaluate the performance of the model.

The optimal unbiased cutoff point of the model was determined using Youden’s index (Youden’s index = sensitivity + specificity − 1). The performance of the classifier was evaluated using metrics such as accuracy, sensitivity, specificity, recall, and F1 score (F1 score is a combination of the model’s precision and recall). The discriminative power of the classifier was assessed using the area under the receiver operating characteristic curve (AUC).

Cross-validation is widely used to reduce bias and overcome overfitting in machine learning when the dataset sample size is small [39]. In this study, we employed the *k*-fold (*k* = 5) cross-validation method to ensure the generalizability of the results. *K*-fold cross-validation divides the dataset into *k* partitions, retains one partition, and uses the remaining *k* − 1 partitions to train/construct the model. The retained partition is then used to evaluate the quality of the trained model. This process is performed k times, with each partition being retained in turn and used to evaluate the model trained using the remaining *k* − 1 partitions.

In addition to the goal of building robust IH prediction models, methods were employed to calculate the importance of individual features to reflect underlying algorithmic decisions, thereby improving clinical acceptability and translation. “Mean Decrease Accuracy” and “Mean Decrease Gini” were used to measure the importance of a feature in RF in discriminating groups. Mean Decrease Accuracy uses permuting “out-of-bag” samples to calculate the importance of a variable to the prediction accuracy of the RF model [40]. Mean Decrease Gini measures the importance of a variable in contributing to the homogeneity of nodes and leaves across all decision trees [40]. The greater the two values, the greater the importance of the features.

**Fig. 1:**
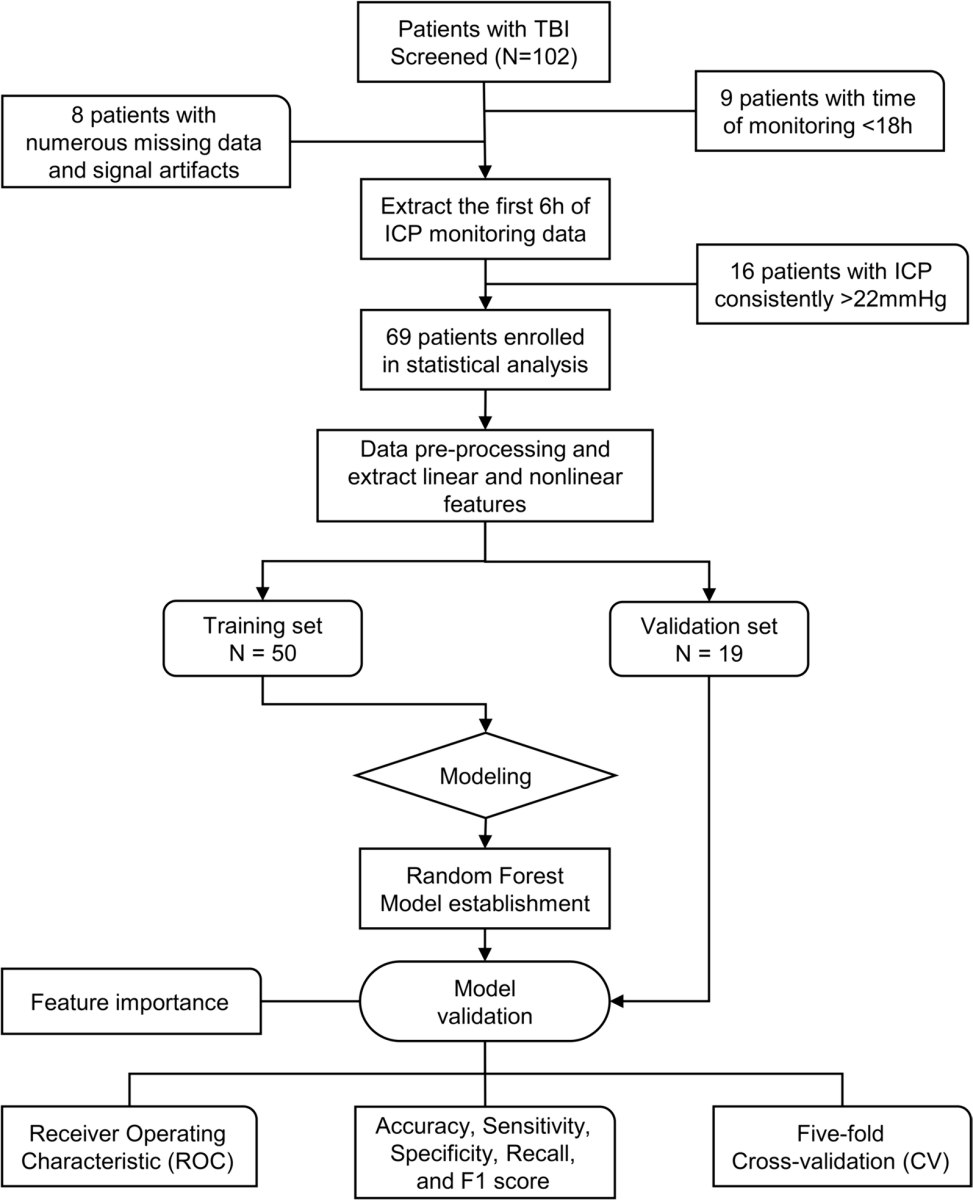
Overview of the study design. *TBI* traumatic brain injury, *ICP* intracranial pressure

The workflow of the study is shown in Fig. 1.

### Statistical analysis

Data were processed and analyzed using R (version 4.3.0) with R Studio (version 2023.03.1 + 446) for Windows as an integrated development environment. Continuous variables that conformed to normal distribution were expressed as the mean (standard deviation), and the *t* test was applied for statistical comparison. Continuous variables that did not conform to normal distribution were expressed as the median and interquartile range (IQR), and the Mann–Whitney *U* test was applied. Categorical variables were expressed as frequencies and percentages, and the Chi-square test was applied. The results were considered significant when *p* < 0.05.

## Results

### Baseline characteristics and features

Sixty-nine patients were included in our analysis, of whom the median age was 54 years (IQR, 45–61years) and 46 patients (66.7%) were male. The median GCS score at admission was 7 (IQR, 6 to 10). The total monitoring time for all patients was 8226.14 h, with a median time of 116.17 h (IQR, 69.65 to 158.87 h) per case. During the monitoring period, 43 patients (62.3%) experienced an IH event, of whom 30 (43%) progressed to SIH. The median time difference between observed IH events and the time of data extraction was 9.83 h (3.35–22.65 h), and for SIH events, it was 11.22 h (4.60–30.24 h).

We confirmed a significant difference in the initial GCS score (7.0 vs. 9.0, *p* = 0.021) between the IH and non-IH groups. Patients who progressed to SIH were significantly older (59.0 vs. 51.0, *p* = 0.042), in addition to having a significantly worse initial GCS score. Regarding ICP-related data, the differences between groups in terms of ICP and PRx were significant, both when IH was distinguished from all patients and when patients who progressed to SIH were distinguished. No significant differences were observed between groups for CPP and RAP. The analysis of nonlinear features indicated that SampEn and Lzc for CPP showed significant differences in distinguishing IH and SIH from all patients. Patient characteristics as well as linear and nonlinear features between groups are shown in Table 1 and the subgroup analysis are shown in Supplementary Tables.

**Table 1:** Patient characteristics as well as linear and nonlinear features between groups

	IH (*n* = 43)	Non-IH (*n* = 26)	*p* value	SIH (*n* = 30)	Non-SIH (*n* = 39)	*p* value
Baseline characteristics						
Age (years)	55.0 [46.0, 61.0]	52.0 [42.0, 59.8]	0.484	59.0 [48.3, 62.0]	51.0 [40.5, 58.5]	0.042
Male (%)	29 (67.4)	17 (65.4)	1	21 (70.0)	25 (64.1)	0.797
Initial GCS	7.0 [6.0, 9.5]	9.0 [7.0, 11.0]	0.021	7.0 [5.3, 9.0]	8.0 [7.0, 11.0]	0.029
Linear features						
ICP (mmHg)	12.7 (5.1)	9.3 (4.3)	0.006	14.1 (4.4)	9.3 (4.6)	< 0.001
CPP (mmHg)	74.3 (9.3)	77.4 (14.1)	0.268	73.4 (9.6)	77.0 (12.4)	0.198
PRx (a.u.)	0.24 (0.18)	0.15 (0.14)	0.027	0.26 (0.19)	0.16 (0.14)	0.022
RAP (a.u.)	0.18 (0.20)	0.20 (0.20)	0.765	0.18 (0.22)	0.20 (0.19)	0.645
Non-linear features						
ICP_SampEn	0.06 [0.05, 0.09]	0.07 [0.04, 0.11]	0.892	0.06 [0.05, 0.09]	0.07 [0.04, 0.10]	0.681
CPP_SampEn	0.37 [0.25, 0.45]	0.25 [0.14, 0.41]	0.009	0.39 [0.31, 0.51]	0.26 [0.17, 0.40]	0.005
ICP_Lzc	0.08 [0.05, 0.10]	0.11 [0.06, 0.14]	0.046	0.08 [0.05, 0.10]	0.09 [0.05, 0.14]	0.185
CPP_Lzc	0.24 (0.11)	0.18 (0.09)	0.024	0.25 (0.12)	0.20 (0.09)	0.049

Categorical variables are expressed as frequencies and percentages; Continuous variables are expressed as the mean (standard deviation) or median and interquartile range (IQR). IH = ICP > 22 mmHg & min > 5; SIH = ICP > 22 mmHg & h > 1

*ICP* intracranial pressure, *CPP* cerebral perfusion pressure, *PRx* pressure reactivity index, *RAP* pressure amplitude correlation index, *SampEn* Sample entropy, *Lzc* Lempel–Ziv complexity

### Forecasting IH and SIH events via random forest

**Fig. 2:**
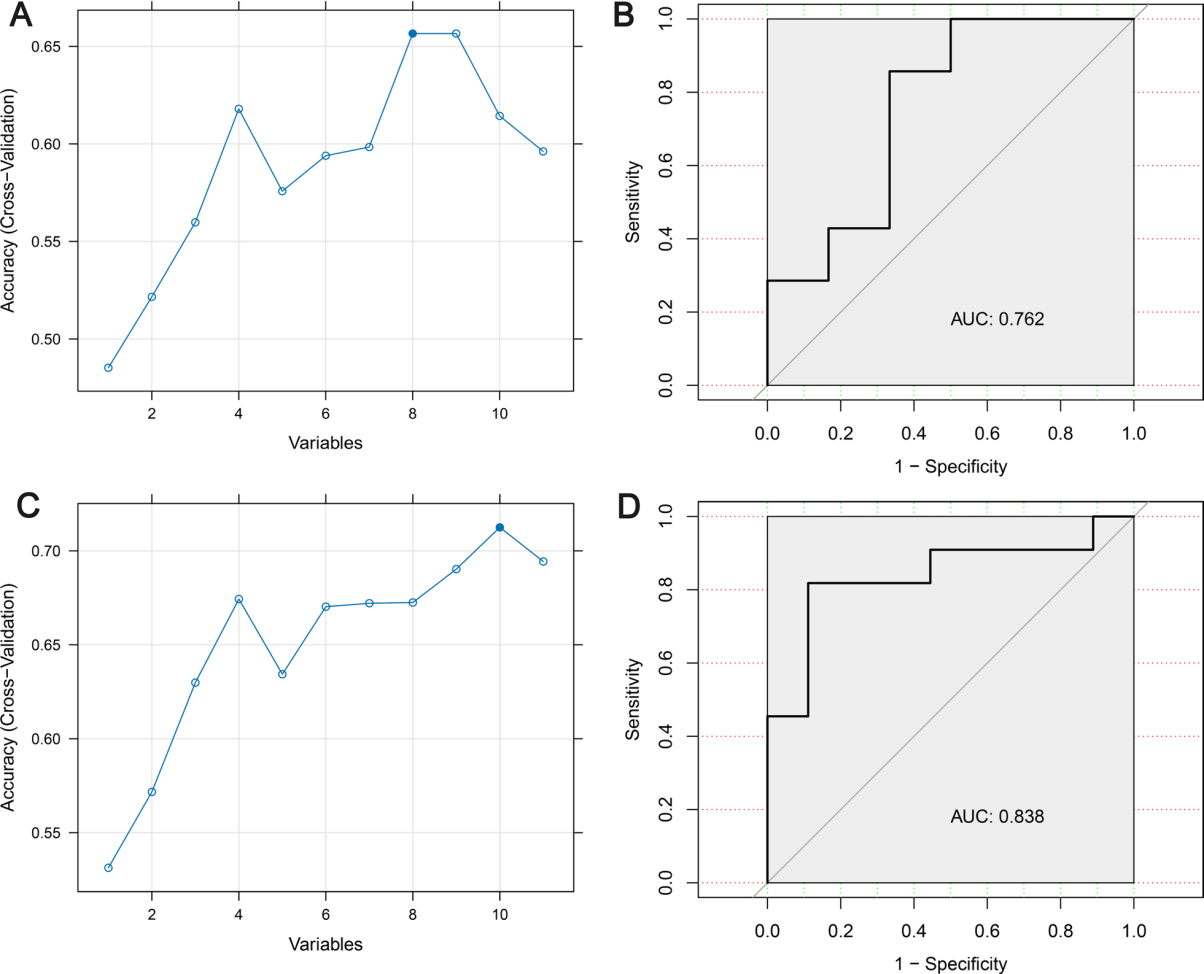
Variable selection process of RFE-CV based on the training set and the receiver operating characteristic (ROC) curve of the RF model for predicting IH and SIH based on the testing set. **A** and **B** are for IH prediction; **C** and **D** are for SIH prediction. *AUC* area under the receiver operating characteristic curve, *RFE* Recursive Feature Elimination, *CV* cross-validation

After performing the FRE-CV feature selection process based on the training set, the following features were incorporated into RF-IH and RF-SIH models: SampEn of CPP, Lzc of CPP, initial GCS, Lzc of ICP, sex, SampEn of ICP, ICP, and CPP for IH model, and ICP, SampEn of CPP, initial GCS, age, PRx, SampEn of ICP, RAP, Lzc of ICP, sex, and Lzc of CPP for SIH model (Table 2, Fig. 2A, C). The optimal features selected by the RFE cross-validation (RFE-CV) and multicollinearity test results are shown in Table 2. The VIF of each feature is less than 5, indicating no multicollinearity between features. In the testing set, the RF-IH model achieved an accuracy of 0.68, sensitivity of 0.43, specificity of 0.83, recall of 0.43, F1 of 0.50, and AUC of 0.76 for predicting IH events (Fig. 2B), while the RF-SIH model had an accuracy of 0.75, sensitivity of 0.91, specificity of 0.56, recall of 0.91, F1 of 0.80, and AUC of 0.84 (Fig. 2D).

**Table 2:** Optimal features selected by the RFE cross-validation (RFE-CV) and multicollinearity test results

RF-IH model	VIF	RF-SIH model	VIF
sex	1.05	sex	1.06
GCSad	1.11	age	1.08
ICP_m	1.39	GCSad	1.24
CPP_m	1.42	ICP_m	1.33
ICP_SampEn	3.04	PRx_m	1.23
CPP_SampEn	2.63	RAP_m	1.53
ICP_Lzc	3.12	ICP_SampEn	2.95
CPP_Lzc	2.87	CPP_SampEn	2.70
–	–	ICP_Lzc	2.84
–	–	CPP_Lzc	2.93

*m* mean value of the variables, *VIF* variance inflation factor, *ICP* intracranial pressure, *CPP* cerebral perfusion pressure, *PRx* pressure reactivity index, *RAP* pressure amplitude correlation index, *SampEn* Sample entropy, *Lzc* Lempel–Ziv complexity

**Fig. 3:**
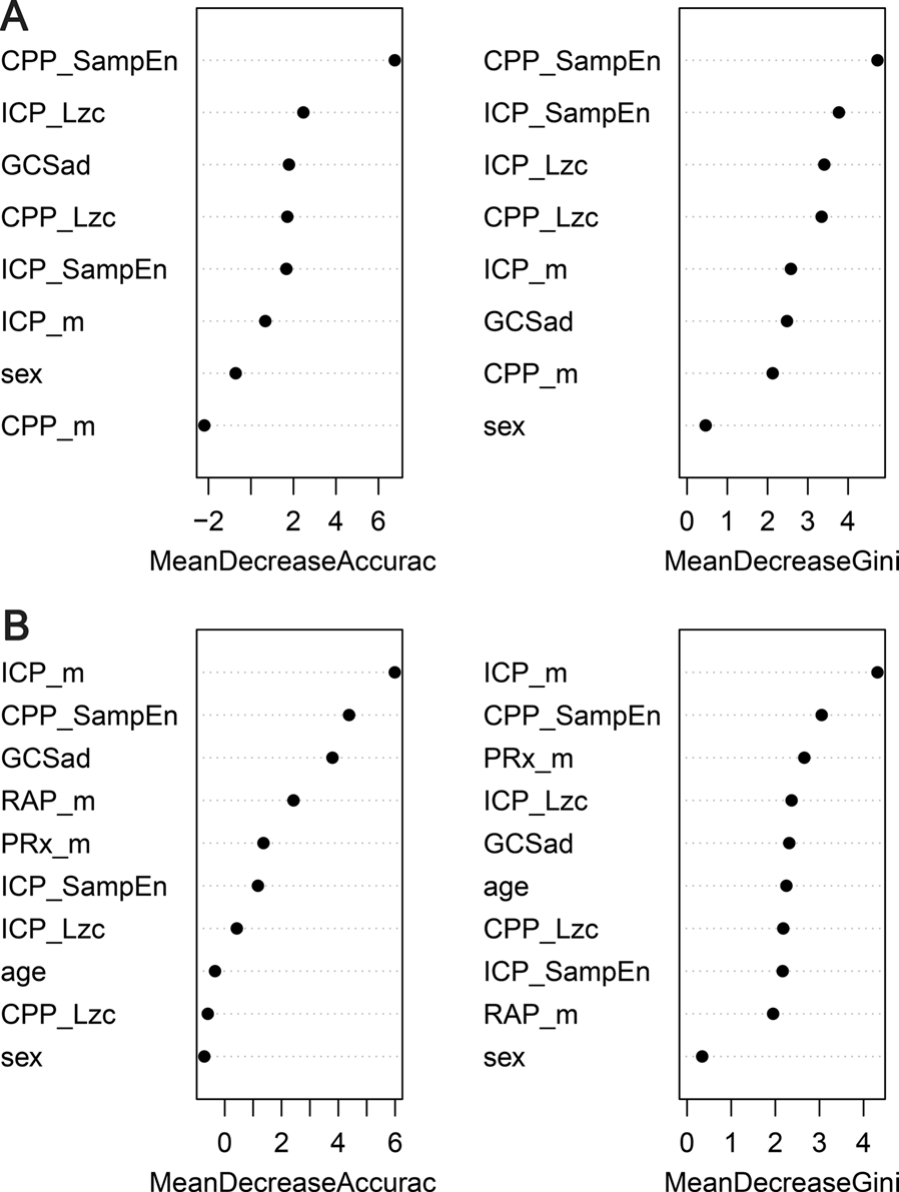
Variable importance measures for each feature of IH and SIH derived from the RF model. **A** Importance ranking of variables in the RF-IH model; **B** importance ranking of variables in the RF-SIH model. *m* mean value of the variables, *ICP* intracranial pressure; *CPP* cerebral perfusion pressure, *PRx* pressure reactivity index, *RAP* pressure amplitude correlation index, *SampEn* Sample entropy, *Lzc* Lempel–Ziv complexity

The features sorted by importance are shown in Fig. 3 to provide an overview of all features contributing to the predicted performance. For distinguishing IH events, the nonlinear features SampEn for CPP and Lzc for ICP demonstrated excellent ability in both importance measures (Fig. 3A). While in predicting SIH events, the ICP mean ranked first in both importance measures, with SampEn for CPP continuing to perform well and ranking second (Fig. 3B).

The cross-validation results of the RF-IH and RF-SIH models are shown in Table 3. The AUCs of the two models were 0.81 (0.07) and 0.79 (0.14), respectively, indicating excellent and stable discriminative abilities. For other evaluation metrics, the RF-SIH model exhibited more excellent performance in terms of accuracy, specificity, and recall.

**Table 3:** Fivefold cross-validation results of RF-IH and RF-SIH models

	RF-IH model	RF-SIH model
Accuracy	0.67 (0.13)	0.80 (0.12)
Sensitivity	0.77 (0.08)	0.70 (0.22)
Specificity	0.51 (0.34)	0.87 (0.09)
Recall	0.75 (0.17)	0.80 (0.13)
F1 score	0.75 (0.08)	0.74 (0.16)
AUC	0.81 (0.07)	0.79 (0.14)

Values are expressed as the mean (standard deviation); AUC is the area under the receiver operating characteristic curve; RF is Random Forest; IH is defined as ICP > 22 mmHg for more than 5 min; SIH is defined as ICP > 22 mmHg for more than 1 h

## Discussion

The present study demonstrates that summarizing linear and nonlinear features and developing random forest models using ICP-related data from the early postoperative period (6 h) can effectively predict subsequent intracranial hypertension events. Two prediction tasks, IH and SIH, were defined, and overall, the developed RF-SIH model performed more excellently. The SampEn of CPP performed well in predicting both IH and SIH, the mean ICP was the strongest predictor of SIH events, and the GCS score showed the highest significance in the demographics. Another advantage of the model developed is the ability to make future IH predictions based on the early phases of ICP monitoring data in the ICU, which is critical for guiding treatment decisions when prognostication is most difficult.

The ICP waveform is generated by the complex interaction between fluids (cerebrospinal fluid and blood) and brain tissue encompassed by a rigid skull [41, 42]. As widely confirmed in the literature, the ICP signal waveform contains much more information than simply the mean ICP value [43]. For example, PRx extracted from the ICP and ABP waveform reflects the autoregulation state of the brain. In this study, we found that individuals who developed future IH or even SIH already showed the deterioration of PRx (consistently > 0.2 or 0.25) during the first 6 h, indicating impaired cerebral autoregulation. RAP did not show a significant difference, which may be attributed to the benefit of early postoperative treatment and surgery that increased the intracranial compensatory space in all patients. In contrast, the PRx was limitedly affected. For instance, studies have shown that PRx does not appear to be substantially affected by decompressive craniectomy [44, 45]. ICP values differed significantly in both group comparisons, suggesting that the ICP of patients with potential IH and SIH already deviated from that of normal patients and approached the upper limit of the normal range. This finding implies a potential benefit of controlling low levels of ICP postoperatively. The difference in CPP was not significant, which may be due to the CPP-oriented regimen at the center, which helped to achieve adequate perfusion pressure in all patients at an early stage.

The complex cerebrospinal fluid–blood–brain tissue system is controlled by interrelated positive and negative feedback systems that dynamically regulate ICP. Sample entropy is a measure of regularity and disorder [23, 46] and Lempel–Ziv complexity characterizes the rate at which new patterns emerge in a time series [33]. These two classical complexity metrics provide comprehensive insights into time series from different perspectives. When the regulatory system of ICP is impaired after TBI, the complexity of ICP may decrease. Previous research has mostly focused on the onset of IH or its relationship to long-term prognosis [47–50]. This study is the first to investigate whether indicators of the complexity of intracranial signals can predict potential IH events. The complexity of ICP did not reveal a generalized difference in the early stages, and only the Lzc of ICP was found to be significantly lower in the IH group and did not further differentiate the SIH subset, suggesting that ICP_Lzc can only differentiate between the occurrence or nonoccurrence of IH. Interestingly, the complexity of the CPP shows generally significant differences, regardless of distinguishing between IH or SIH events. Patients with potential IH showed higher CPP complexity, indicating unstable CPP and impaired brain autoregulation compared to normal patients. This is consistent with impaired PRx but needs to be interpreted with caution.

The development of an IH early warning system has been introduced previously. Various research groups are working on developing technologies to enable preventive management of IH to improve patient outcomes [16, 51–55]. Current mainstream ICP waveform-based algorithms are still plagued by the prediction window that is too short to provide clinicians with enough time to intervene ahead of time [14]. This study is based on data from patients in the early postoperative period, aiming to obtain an overview of the patient's early intracranial condition and predict the occurrence of IH in the following days. This gives clinicians enough time to respond, for example, by implementing more aggressive therapeutic measures for certain patients, rather than waiting for the onset of IH and intervening reactively. The developed model considers the baseline clinical characteristics, the linear features, and the complexity of the signal nonlinear features as compensation. The performance is excellent, and a stable performance is obtained in the cross-validation. Overall, the model performs better in predicting the SIH population in the center with higher accuracy and recall values.

Performance and interpretability in clinical systems are critical to support clinical decision-making [56]. Therefore, visualizing the features that influence the RF model’s prediction is essential. The SampEn of CPP performs well in predicting IH or SIH, indicating that patients with potential IH tend to have greater CPP fluctuations in the early phases. As with the previous statistical tests, this was an unexpected finding. This suggests that focusing on fluctuations in a patient’s CPP might be more valuable than ICP. Focusing on the fluctuations or complexity of physiologic indicators is equally important as examining their values. Another noteworthy finding is that the average ICP value was the strongest predictor of SIH events, consistent with the results of previous statistical analyses. This suggests that personalized ICP thresholds are essential for identifying high-risk populations, a challenge that has plagued researchers. For the clinical characteristics, due to the small sample size of a single center, the inclusion of too many characteristics would lead to overfitting of the model. Therefore, the study focused on essential demographics including age, sex, and GCS score representing the severity of TBI. Overall, the GCS score showed the highest importance in demographics, while gender showed the least importance.

Several limitations of the study must be noted. First, this is an exploratory study based on a single center with a limited sample size, which reduces the generalizability of the findings, and further validation is needed despite the use of cross-validation. Second, this was an observational study that could not control for the effects of clinical interventions (e.g., medication and/or ventilator weaning). Even though all patients were monitored in a single neuro-intensive care unit and therapeutic interventions were standardized, variations were inevitable. Third, patients with persistent ICP greater than 22 mmHg were excluded from this study in advance, which might impact the representativeness of the patient population. Nevertheless, the focus of this study was on predicting IH events that have not yet occurred, and it is believed that guidance for clinical intervention is a more important consideration.

## Conclusions

In this study, we extracted linear and nonlinear features based on ICP-related data in the early postoperative period following traumatic brain injury and combined them with baseline features to effectively predict subsequent IH, especially SIH events, using an interpretable random forest technique. Although this result is prior to external validation, we believe that it provides a potentially practical predictive pathway for researchers and clinicians. In the future, this framework may assist in triaging patients requiring more intensive neurologic treatment at an early stage.

### Supplementary Information

Supplementary Material 1.

## Data Availability

The data sets used and/or analysed during the current study are available from the corresponding author on reasonable request.

## Abbreviations

IH: Intracranial hypertension
SIH: Severe intracranial hypertension
TBI: Traumatic brain injury
RF: Random Forest
ICP: Intracranial pressure
AUC: Area under the receiver operating characteristic curve
CPP: Cerebral perfusion pressure
PRx: Pressure reactivity index
RAP: Pressure amplitude correlation index
ICU: Intensive care unit
GCS: Glasgow Coma Scale
ABP: Arterial blood pressure
MAP: Mean arterial pressure
SampEn: Sample entropy
Lzc: Lempel–Ziv complexity
BTF: Brain Trauma Foundation
RFE: Recursive Feature Elimination
CV: Cross validation
IQR: Interquartile range
ROC: Receiver operating characteristic
