# Supplementary material for: Random forest-based prediction of intracranial hypertension in patients with traumatic brain injury
Source: Intensive Care Med Exp. 2024 Jul 2;12:58. doi: 10.1186/s40635-024-00643-6 (PMC11219663; doi:10.1186/s40635-024-00643-6)
Supplement: Supplementary file 1 — Supplementary Material 1. [file 40635_2024_643_MOESM1_ESM.docx]

**Supplementary table 1.** Patient characteristics as well as linear and nonlinear characteristics between the non-IH, IH, and SIH groups.

|  | non-IH (n=26) | IH (n=13) | SIH (n=30) | p-value |
| --- | --- | --- | --- | --- |
| **Baseline characteristics** |  |  |  |  |
| Age (years) | 52.0 [42.0, 59.8] | 49.0 [41.0, 55.0] | 59.0 [48.3, 62.0] | 0.114 |
| Male (%) | 17 (65.4) | 8 (61.5) | 21 (70.0) | 0.851 |
| Initial GCS | 9.0 [7.0, 11.0] | 7.0 [7.0, 10.0] | 7.0 [5.3, 9.0] | 0.091 |
| **Linear features** |  |  |  |  |
| ICP (mmHg) | 9.3 (4.2) | 9.3 (5.3) | 14.1 (4.4) | <0.0001 |
| CPP (mmHg) | 77.4 (14.1) | 76.2 (8.6) | 73.4 (9.6) | 0.418 |
| PRx (a.u.) | 0.15 (0.14) | 0.20 (0.15) | 0.26 (0.19) | 0.049 |
| RAP (a.u.) | 0.20 (0.20) | 0.20 (0.17) | 0.18 (0.22) | 0.900 |
| **Non-linear features** |  |  |  |  |
| ICP_SampEn | 0.07 [0.04, 0.11] | 0.05 [0.05, 0.08] | 0.06 [0.05, 0.09] | 0.492 |
| CPP_SampEn | 0.25 [0.14, 0.41] | 0.33 [0.20, 0.39] | 0.39 [0.31, 0.51] | 0.011 |
| ICP_Lzc | 0.11 [0.06, 0.14] | 0.08 [0.05, 0.09] | 0.08 [0.05, 0.10] | 0.356 |
| CPP_Lzc | 0.18 (0.09) | 0.23(0.07) | 0.25 (0.12) | 0.066 |

Categorical variables are expressed as frequencies and percentages; Continuous variables are expressed as the mean (standard deviation) or median and interquartile range (IQR). IH = ICP>22 mmHg & minutes>5; SIH = ICP>22 mmHg & hour>1. ICP is intracranial pressure; CPP is cerebral perfusion pressure; PRx is pressure reactivity index; RAP is pressure amplitude correlation index; SampEn is Sample entropy; Lzc is Lempel-Ziv complexity.

**Supplementary table 2.** Bonferroni test results for intergroup comparisons.

|  | ICP (mmHg) | PRx (a.u.) | CPP_SampEn |
| --- | --- | --- | --- |
| non-IH group vs. IH group | 1 | 1 | 1 |
| non-IH group vs. SIH group | <0.0001 | 0.044 | 0.01 |
| IH group vs. SIH group | 0.006 | 0.834 | 0.246 |

IH = ICP>22 mmHg & minutes>5; SIH = ICP>22 mmHg & hour>1. ICP is intracranial pressure; CPP is cerebral perfusion pressure; PRx is pressure reactivity index; SampEn is Sample entropy.
